# Efficient Transduction of Primary Vascular Cells by the Rare Adenovirus Serotype 49 Vector

**DOI:** 10.1089/hum.2015.019

**Published:** 2015-03-11

**Authors:** Rachel S. Dakin, Alan L. Parker, Christian Delles, Stuart A. Nicklin, Andrew H. Baker

**Affiliations:** ^1^ Institute of Cardiovascular and Medical Sciences, BHF Glasgow Cardiovascular Research Centre, University of Glasgow, Glasgow G12 8TA, United Kingdom.

## Abstract

Neointima formation and vascular remodeling through vascular smooth muscle cell migration and proliferation can limit the long-term success of coronary interventions, for example, in coronary artery bypass grafting (CABG). *Ex vivo* gene therapy has the potential to reduce unnecessary cell proliferation and limit neointima formation in vascular pathologies. To date, the species C adenovirus serotype 5 has been commonly used for preclinical gene therapy; however, its suitability is potentially limited by relatively poor tropism for vascular cells and high levels of preexisting immunity in the population. To avoid these limitations, novel species of adenovirus are being tested; here we investigate the potential of adenovirus 49 (Ad49) for use in gene therapy. Transduction of primary human vascular cells by a range of adenovirus serotypes was assessed; Ad49 demonstrated highest transduction of both vascular smooth muscle and endothelial cells. Gene transfer with Ad49 in vascular smooth muscle and endothelial cells was possible following short exposure times (<1 hr) and with low MOI, which is clinically relevant. *Ex vivo* delivery to surplus CABG tissue showed efficient gene transfer with Ad49, consistent with the *in vitro* findings. Luminal infusion of Ad49GFP into intact CABG samples *ex vivo* resulted in efficient vessel transduction. In addition, no seroprevalence rates to Ad49 were observed in a Scottish cohort of patients from cardiovascular clinics, thus circumventing issues with preexisting immunity. Our results show that Ad49 has tropism for vascular cells *in vitro* and *ex vivo* and demonstrate that Ad49 may be an improved vector for local vascular gene therapy compared with current alternatives.

## Introduction

Cardiovascular disease remains one of the leading causes of mortality worldwide.^1^ Use of gene therapy, targeting myocardium and vascular cells, remains a promising strategy for treatment of a variety of cardiovascular diseases. Efficient gene transfer to cardiomyocytes using adeno-associated virus (AAV) vectors has been shown in many preclinical models (reviewed in ref.^2^), and promising results have been reported in the phase 2 CUPID clinical trial overexpressing sarcoplasmic reticulum Ca^2+^-ATPase (SERCA2a) via AAV-1 in patients with advanced heart failure.^3^ Outside the myocardium, targeting vascular cells for gene therapy could be beneficial in reducing both cardiovascular diseases, for example, angiogenesis associated with ischemic disease and pathological vascular remodeling associated with vein graft failure. Coronary revascularization procedures are some of the most common major medical surgeries. In coronary artery bypass grafting (CABG), a peripheral section of vasculature, usually saphenous vein is used to bypass the occluded vessel in the coronary circulation. Following the procedure, the vessel undergoes a period of remodeling to adapt to the increased blood flow and pressure in the arterial circulation. However, excessive vascular smooth muscle cell (VSMC) migration and proliferation and damaged endothelium cause neointima formation and can limit the long-term success of the graft; studies report that >25% of grafts have reduced patency after 4 years and up to 50% at 10 years.^4^

Reduction of neointima formation by gene delivery to the vasculature is an attractive proposition to improve long-term patency of CABG procedures and reduce the need for repeat surgery. Unlike cardiomyocytes, only low levels of transduction are possible in VSMCs or endothelial cells (ECs) using AAVs.^5,6^ However, preclinical studies using adenovirus vectors have shown that gene delivery to the vasculature can prevent neointima formation.^7,8^ Expression of therapeutic proteins, including endothelial nitric oxide synthase (eNOS),^9^ inducible nitric oxide synthase (iNOS),^10^ tissue inhibitors of metalloproteinases 1–3 (TIMPs 1–3),^7,11,12^ p53,^13^ and Nogo-B,^14^ has been shown to enhance EC regeneration and/or reduce VSMC proliferation and migration, in turn reducing neointima formation, in animal models of CABG. In fact, CABG surgery may be particularly suited to gene therapy as the clinical setting facilitates *ex vivo* gene transfer to the vein before implantation. To date, the majority of gene therapy strategies have used adenovirus serotype 5 (Ad5) as the gene delivery vector. However, Ad5 transduces vascular cells relatively poorly and high multiplicities of infection are required to achieve therapeutic levels of transgene expression.^15,16^

Previous analysis of receptor expression on VSMCs and ECs has shown only low levels of coxsackie and adenovirus receptor (CAR),^17^ the primary Ad5 receptor, and no detectable expression in intact human vessels.^18^ Additionally, in CABG, the time available for *ex vivo* gene transfer before grafting is short, potentially less than 30 min, further limiting the gene delivery possible with Ad5. An added clinical consideration of Ad5 use is the common prevalence of preexisting neutralizing antibodies in the population,^19^ up to 80% seroprevalence in some studies,^20^ which may reduce the efficacy of transgene expression,^21^ although the effect of preexisting neutralizing antibodies on adenovirus transduction in the CABG setting is unknown as gene transfer occurs *ex vivo*. Together, these limitations of Ad5 suggest that, while the vector may be suitable for human CABG gene therapy, alternative vectors might provide improved opportunities.

To increase transgene expression in the vasculature, pseudotyping of Ad5 has been shown to be effective.^22^ Several subgroup B adenoviruses, including Ad35, use CD46 as a receptor,^23^ which is relatively highly expressed on VSMCs, and chimeric Ad5/Ad35 viruses show more efficient gene transfer to the vasculature than Ad5.^17^ An alternative strategy is exploitation of the natural tropism of novel Ad serotypes. Subgroup D adenoviruses vary in receptor use and tropism; some infect cells by binding CAR, while others use CD46^24^ or sialic acid.^25^ However, the receptors for many subgroup D adenoviruses have not been identified, and therefore they may provide new vectors for gene therapy. An additional clinical benefit is that species D adenoviruses are reported to have low seroprevalence rates.^24^ Here we evaluated a panel of adenoviruses and highlight the efficacy of Ad49 as a vector for vascular gene therapy.

## Materials and Methods

### Cells and tissue

PER.C6 cells^26^ (kind gift from Jerome Custers, Crucell) and HEK293 cells (human embryonic kidney: ATCC CRL-1573) were used for viral propagation and cultured in Dulbecco's modified Eagle's medium (DMEM; Invitrogen) with 2 m*M* L-glutamine (Invitrogen) and 10% fetal calf serum (FCS; PAA Laboratories) with the addition of 10 m*M* MgCl_2_ (Sigma-Aldrich) for PER.C6 cells. Hep G2 (hepatocellular carcinoma: ATCC HB-8065), A549 (human lung carcinoma: ATCC CCL-185), and SKOV3 (human ovarian carcinoma: ATCC HTB-77) cells were cultured in minimal essential medium (MEM) or RPMI-1640 medium (Invitrogen), with 2 m*M* L-glutamine, 10% FCS, and 1 m*M* sodium pyruvate (Sigma-Aldrich). Cells were maintained at 37°C and 5% CO_2_ with the exception of PER.C6 maintained in 10% CO_2_. Human saphenous vein segments were obtained from patients, who gave informed consent, undergoing CABG. Ethics permission was obtained from the West of Scotland Research Ethics Committee 4 (reference number 10/S0704/60). Serum samples were obtained, with consent, from patients attending cardiovascular disease clinics with ethics permission from the West Glasgow Ethics Committee 1 (reference number S/0703/110).

### Smooth muscle and EC isolation and culturing

VSMCs were isolated from human saphenous vein segments using the explant technique as previously described^27^ and cultured in SMC growth media 2 (PromoCell) with addition of provided supplement mix, 10% FCS, and 2 m*M* L-glutamine. ECs were isolated from human saphenous vein segments by collagenase digestion as previously described and cultured in Large Vessel Endothelial Cell Basal Medium (TCS CellWorks) with provided supplements and 20% FCS. Primary cell lines were used between passage 3 and 6.

### Recombinant adenoviral vectors

All recombinant adenoviruses described in this article were kind gifts from Jerome Custers (Crucell); Ad5, Ad26, Ad35, Ad48, and Ad49 are replication-incompetent E1/E3 deleted vectors constructed as described previously.^28,29^ Viruses were propagated in HEK293 or PER.C6, E1-complementing cell lines and purified using CsCl gradients. Viral recovery was quantified by microBCA assay (ThermoFisher) assuming that 1 μg protein=4×10^9^ viral particles (vp) and confirmed by nanosight measurement (Nanosight). Infectious units (pfu) were quantified by end-point dilution plaque assay.^30^

### Transduction assays and neutralization assays

Cells were plated in 96-well format, 1×10^4^ cells/well, and incubated overnight. Cells were infected with 1000, 2500, 5000, or 10,000 vp/cell in 50μl serum-free media, ±hFX (8 μg/ml) where stated. Cells were incubated with virus for 3 hr at 37°C, unless otherwise stated; washed with PBS; and maintained in standard medium until harvesting with reporter lysis buffer (RLB; Promega) 48 hr postinfection. Luciferase activity was measured in cell lysates using a luciferase assay (Promega) and protein concentrations were calculated by BCA (ThermoFisher) assays were measured using a Wallac Victor2 plate reader (PerkinElmer) and values expressed as relative light units (RLU)/mg of protein. For serum neutralization studies, transduction of Hep G2 cells was carried out as above in the presence of 2.5% serum as previously been shown to be optimum.^31^ Samples were considered neutralizing if greater than 90% reduction in transduction was seen in comparison to no serum control. To determine GFP expression, cells were detached from the culture vessel with 1xTE, washed, and quantified using a flow-assisted cell sorter (FACS) Canto II flow cytometer (Becton Dickinson) and FACS DIVA software. Viable cells were gated by their FSC/SSC profiles, with a minimum of 10,000 gated events analyzed per sample. Results are expressed as percentage of positively stained cells per sample, from three independent samples analyzed in triplicate.

### *Ex vivo* transduction of CABG tissue

Human saphenous vein tissue was trimmed to remove excess periadventitial fat and divided into equal (∼5 mm) sections before incubation with adenovirus: 1×10^9^ vp or PBS in 500 μl serum-free media for 1 hr at 37°C. Tissue sections were washed and cultured for a further 48 hr in SMC media. Luciferase activity was analyzed using bioluminescence quantification imaging (IVIS Spectrum; Caliper Life Sciences) quantified as average radiance (photons per second per centimeter squared per steradian). Human saphenous veins were transduced by luminal delivery of adenoviruses as previously described.^11^ Four- to six-centimeter segments of vein were cannulated, and the luminal surface was exposed to adenoviral vectors at 1×10^10^ pfu/ml at physiological pressure for 1 hr. Segments of vein were cultured for 3 days in DMEM containing 30% FCS and antibiotics.

### Immunohistochemistry

Immunohistochemistry was performed on 4 μm paraformaldehyde fixed sections. Following rehydration, heat-induced antigen retrieval was performed with 10 m*M* sodium citrate buffer (pH 6) and sections were blocked in 15% goat serum before incubation with primary antibodies: chicken anti-gfp (Abcam), mouse anti-smooth muscle actin (Dako), and rabbit anti-von Willebrand factor (Dako). Fluorescent secondary antibodies were used for detection, goat anti-chicken 488, anti-rabbit 546, and anti-mouse 647 (Invitrogen), and slides were mounted with ProLong Gold Antifade Reagent with DAPI (Invitrogen). Images were acquired using a Zeiss LSM510 confocal imaging system (Carl Zeiss).

### Statistics

*In vitro* data presented are representative from a minimum of three separate experiments with at least three experimental replicates per group unless otherwise stated. Transduction graphs are displayed with a log scale as appropriate, and for these experiments data were log transformed before analysis. Statistical significance was calculated using ANOVA or Student's *t*-test; *p*<0.05 was considered statistically significant.

## Results

### Ad49 tropism for human vascular cells *in vitro*

To initially evaluate vector tropism for vascular cells, the transduction efficiency of four serotypes of adenovirus was evaluated in primary VSMCs and ECs, in comparison to the commonly used vector Ad5. Previous reports have demonstrated relatively high transduction of vascular cells by Ad35,^17^ therefore acting as a positive control for efficient transduction in this study. The other three Ad serotypes chosen belonged to subgroup D and have not been investigated in the terms of vascular cell transduction. Additionally, low seroprevalence to these Ad serotypes has been found^24,32^ in serum samples from sub-Saharan Africa.

**FIG. 1.**
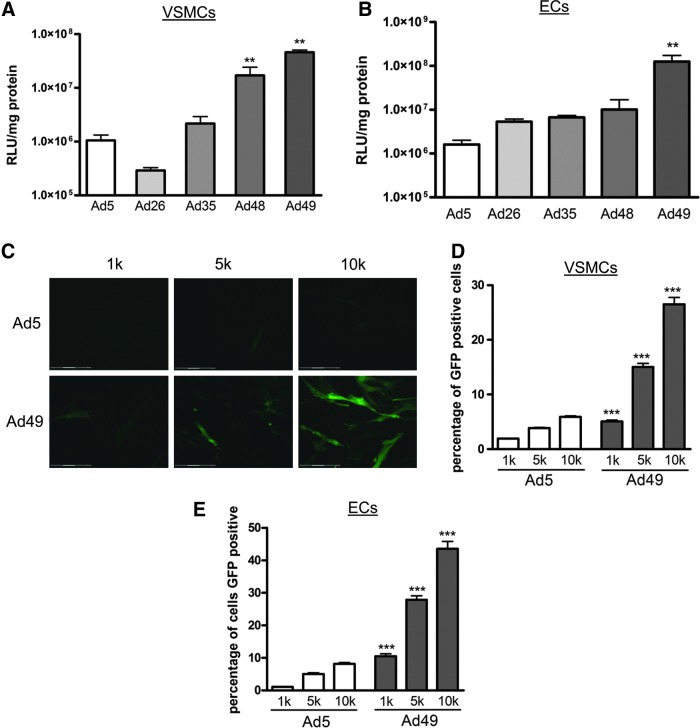
Transduction of primary vascular cells. VSMCs **(A)** and ECs **(B)** were transduced with 10,000 vp/cell of adenovirus for 3 hr at 37°C. Cells were cultured for a further 48 hr before analysis of luciferase transgene expression was performed. Representative images (20×) of VSMCs following transduction of 1,000 (1k), 5,000 (5k), or 10,000 (10k) vp/cell Ad5 or Ad49–GFP are shown in **(C)**. FACS analysis of GFP expression 48 hr posttransduction in hVSMCS **(D)** and ECs **(E)**. Data are mean+SE compared with Ad5 by one-way ANOVA with Dunnett's *post-hoc* analysis or Student's *t*-test; **p<0.01, ***p<0.001. Ad5, adenovirus serotype 5; ECs, endothelial cells; vp, viral particles; VSMCs, vascular smooth muscle cell.

First, transduction efficiency was assessed using vectors expressing luciferase. In comparison to Ad5, VSMCs transduced with Ad48 and Ad49 had significantly higher luciferase activity (Fig. 1A). Similarly, Ad49 also had the highest luciferase activity following infection of ECs (Fig. 1B). To enable visualization of transgene expression in vascular cells, an Ad49 vector expressing GFP was generated. VSMCs and ECs were transduced with a range of viral titers and fluorescence assessed microscopically, and representative images of VSMCs are shown in Fig. 1C. GFP expression was further analyzed using FACS, and VSMCs (Fig. 1D) and ECs (Fig. 1E) transduced with Ad49 had higher GFP expression compared with Ad5. Collectively, these data demonstrate that Ad49 can efficiently transduce primary human vascular cells.

To investigate more general selectivity, Ad49 gene transfer was assessed in other cell lines with different receptor profiles (Supplementary Fig. S1; Supplementary Data are available online at www.liebertpub.com/hum).

### Transduction of VSMC and ECs in short time periods

**FIG. 2.**
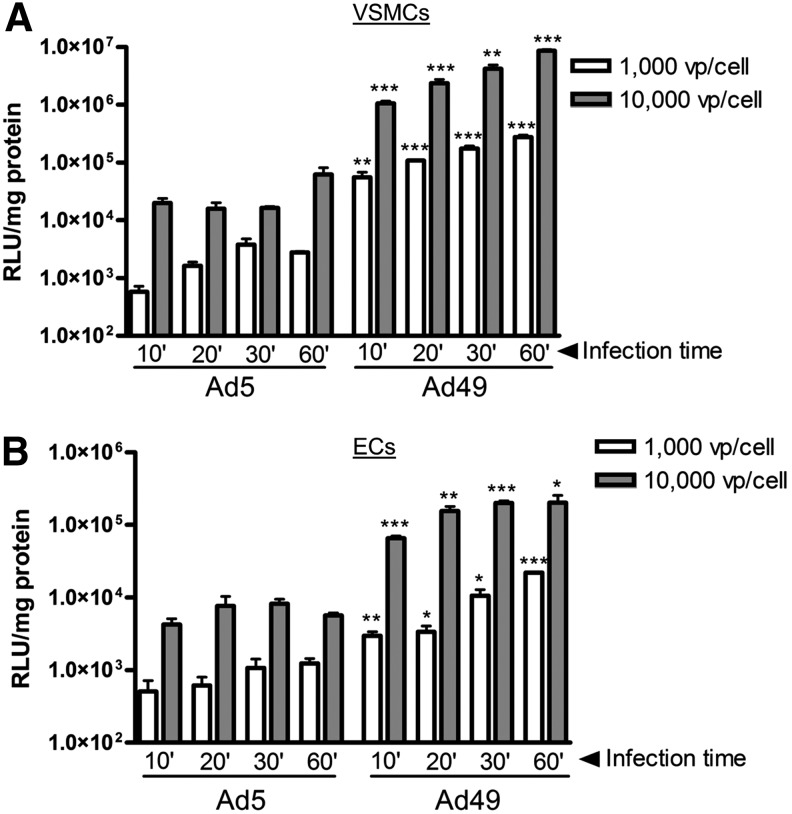
Transduction of cells with short exposure times. VSMCs **(A)** and ECs **(B)** were infected with 1,000 and 10,000 vp/cell of adenovirus for 10–60 min. Cells were cultured for a further 48 hr before analysis of luciferase transgene expression was performed. Data are mean+SE compared with equivalent Ad5 with Student's *t*-test; *p<0.05, **p<0.01, ***p<0.001.

In the context of CABG surgery, the time available for gene transfer is limited, and therefore rapid and efficient transduction is important. To investigate the potential for Ad49 to transduce cells following minimal, clinically relevant contact times, exposure of Ad5 or Ad49 to VSMCs and ECs was reduced to between 10 and 60 min. In comparison to Ad5, higher luciferase activity was detected in VSMCs (Fig. 2A) and ECs (Fig. 2B) transduced with Ad49 at all time points tested, down to just 10 min exposure. Furthermore, efficient gene transfer was possible when both low titers of 1,000 vp/cell and high titers of 10,000 vp/cell were used for short incubation times (Fig. 2A and B).

### *Ex vivo* gene transfer to intact saphenous vein

**FIG. 3.**
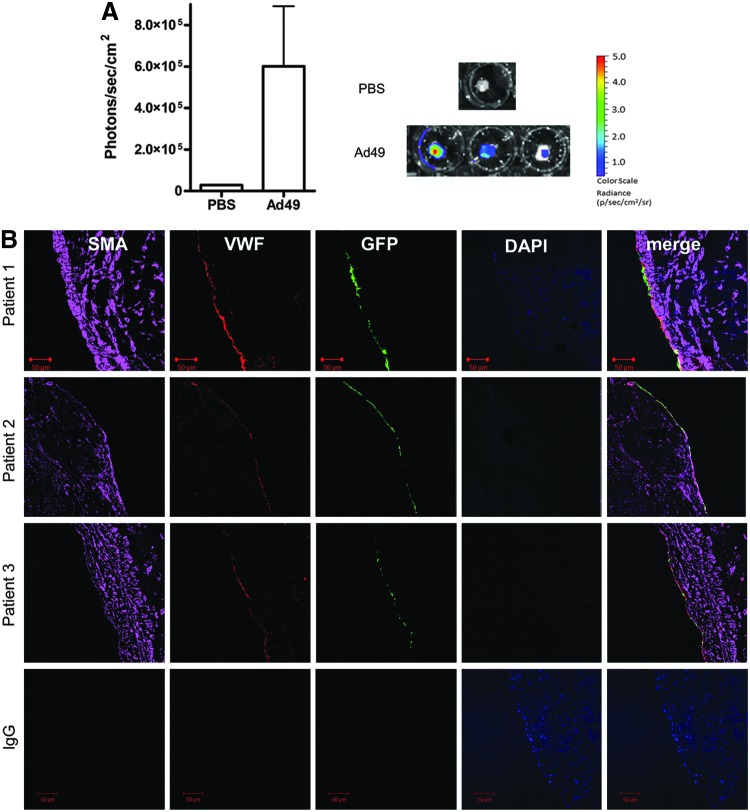
*Ex vivo* transduction of human coronary artery bypass graft tissue. Surplus coronary artery bypass grafting (CABG) tissue was cut into equal sections and incubated with 1×10^9^ vp Ad49LUC or PBS for 1 hr at 37°C. Media was replaced with 20% and samples were cultured for a further 48 hr. Luciferase activity was analyzed using the IVIS system and quantified as average radiance **(A)**. Data are mean+SEM. Luminal dwell of Ad49GFP (1×10^10^ pfu/ml) was performed for 1 hr at room temperature on surplus saphenous vein. Vessels were opened longitudinally and cultured for 72 hr. GFP expression was visualized using immunohistochemistry **(B)**. Sections of 4 μm were stained with antibodies against α-smooth muscle actin (SMA, purple), von Willebrand factor (VWF, red), and GFP (green) or relevant IgG controls, and visualized using confocal microscopy at 10×.

The above data demonstrate Ad49 as an efficient vector for gene transfer to vascular cells *in vitro*. To further investigate the potential for clinical use, transduction of surplus CABG tissue was undertaken. First, whole vessels were incubated with Ad vectors and luciferase activity was quantified using an IVIS imaging system. Consistent with the *in vitro* data, luciferase activity was detected in tissue exposed to Ad49 at significantly increased levels to control (Fig. 3A). Follow-up studies exposing only the luminal surface of the tissue to Ad49GFP were performed in CABG samples from three patients. Cells positive for GFP co-localized with those stained for von Willebrand factor, a marker of ECs (Fig. 3B). Sections were stained throughout the length of vein exposed to Ad49GFP, and positive GFP staining was detected in ECs throughout the vessel.

### Preexisting immunity to Ad49

**FIG. 4.**
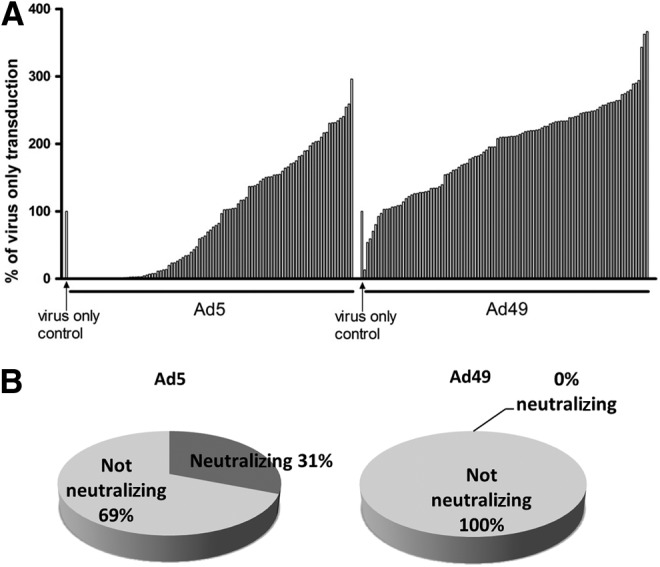
Preexisting immunity to Ad49 in patients undergoing CABG. Transduction assays were carried out in the presence of serum samples from 103 patients from Glasgow Cardiovascular Clinics, and each bar represents 1 patient **(A)**. Samples with 90% or greater reduction in adenovirus-mediated luciferase expression, compared with virus only control, were considered neutralizing **(B)**.

The use of adenovirus vectors in a clinical setting may be limited by preexisting immunity,^33^ depending on the route of administration and target tissue, although this may not be problematic where *ex vivo* gene transfer is being utilized. To determine the seroprevalence of Ad49 in a clinically relevant population, serum samples were collected from 103 patients in Glasgow cardiovascular clinics. Presence of neutralizing antibodies was determined by preincubating Ad5 or Ad49 with 2.5% serum, recognized as an optimal dilution,^24,31^ before transduction of HepG2 cells (Fig. 4A). Samples were considered neutralizing if transduction was reduced by 90% compared with virus alone, previously shown to be ideal for studying neutralization patterns of Ads.^22,34^ Ad5 was neutralized by 31% (32/103) of serum samples, while none of the 103 serum samples reduced Ad49 cell transduction by 90% (Fig. 4B). Further analysis found that 1 serum sample could reduce Ad49 transduction by 50%, while an additional 16 samples could reduce Ad5 transduction to between 10% and 50% of control.

## Discussion

The aim of this study was to identify an Ad serotype suitable for use as a vascular gene therapy vector: assessing efficacy *in vitro* and *ex vivo* and determining any seroprevalence in the population that may limit clinical use. For the first time we report the natural tropism of Ad49, a species D adenovirus, for vascular cells demonstrating efficient gene transfer in primary vascular cells and human CABG tissues.

Previous vascular gene therapy studies have used high concentrations of Ad5 for gene transfer. Ad5 transduces cells through engagement with CAR and further internalization using integrins.^35,36^ The limited expression of CAR on vascular cells presumably leads to the requirement for high concentrations of Ad5. The *in vitro* results presented here demonstrate that Ad49 is able to transduce both VSMCs and ECs, resulting in higher reporter gene activity than Ad5. We demonstrated that gene transfer with an alternative reporter gene, *GFP*, was more efficient with Ad49 than Ad5 over a range of viral concentrations. Ad35 and pseudotyping of Ad5 with Ad35 fiber have previously been proposed as efficient vascular gene therapy vectors because of high expression of their receptor CD46 on vascular cells.^17^ Interestingly, these results suggest that Ad49 can transduce vascular cells with greater efficiency than Ad35 and that lower concentrations of vector than have been previously used may be sufficient *in vivo*.

Furthermore, we tested the efficacy of Ad49 following short exposure times on cells because of the limited clinical time available for gene transfer. In comparison to Ad5, luciferase activity was 100 times higher following Ad49 transduction of VSMC and ECs and still efficient with low multiplicity of infection and only 10 min incubation with cells. The transduction efficiency of Ad49 in the cell lines, A549, SKOV3, and Hep G2, was similar or less than that of Ad5. This further supports the superior efficiency of the vector in vascular cells compared with Ad5, which is not evident in all cell types. It will be important to determine the primary receptor used by Ad49. The *in vitro* data led us to speculate that Ad49 could be a useful vector for vascular gene transfer, using lower concentrations and incubation times than is optimal with Ad5.

To confirm the clinical relevance of the Ad49 vector, further gene transfer experiments were carried out *ex vivo* in CABG tissue. While primary cells are a good tool for modeling *in vitro*, their phenotype can alter in culture in isolation from normal neighboring cell types.^37^ First, whole sections of CABG tissue were incubated with Ad49, confirming that efficient vascular transduction of intact tissues was possible. To recreate the clinical situation and determine exactly which cell types were being transduced *ex vivo*, we delivered Ad49 to the lumen of saphenous vein. The luminal surface of the vessel will experience the dramatic changes in flow and pressure following grafting into the arterial circulation and as such these cells may be the optimal clinical target for preventing neointima formation. Efficient gene transfer of GFP was detected in samples from three different patients. Interestingly, transgene expression co-localized with positive staining for the EC marker von Willebrand factor. This is consistent with the transduction data in primary cultured ECs from similar samples; however, no gene transfer was detected in the medial smooth muscle cell layer. Previous reports have shown Ad5 gene transfer to VSMC.^12^ The reason for the differences in cell specificity *ex vivo* is currently unclear but may be because of receptor expression distribution and levels and/or differences in surgical approaches to graft harvest and treatment. It has been previously suggested that Ad49 can bind to CD46,^28^ which is highly expressed on ECs.^17^ Additionally, over the last decade surgeons have adopted a no-touch technique for harvest of saphenous veins that has been shown to reduce SMC activation and damage to ECs.^38^ The change in harvest method, maintaining a complete endothelial layer, could contribute to the distribution of infected cells.

Gene transfer to ECs is clinically relevant as, in addition to control of VSMC proliferation, ECs often get damaged during vascular procedures and re-establishing a healthy endothelium can reduce neointima formation.^39–41^ Despite the new surgical techniques designed to preserve the endothelium, CABG failure is still a clinical problem. Delivery of eNOS^42^ and vascular endothelial growth factor (VEGF)^43^ to ECs has been shown to reduce neointima formation in animal models. Furthermore, delivery of Ad49 vectors encoding secreted transgenes such as p53^13^ or A20^44^ could be used to reduce the VSMC proliferation associated with neointima formation. EC gene therapy is also beneficial in other disease settings: to promote revascularization of ischemic tissue and reduced angiogenesis in cancer. Efficient gene transfer has thus far been limited in angiogenic gene therapy (reviewed in ref.^45^), Ad49 may also be a useful vector in this setting, and further research is required.

Clinical use of viral vectors can be limited by preexisting immunity. In the CUPID clinical trial, almost 50% of patients had to be excluded because of AAV-neutralizing antibody titers of >1/2.^46^ Preexisting immunity to Ad5 has been demonstrated in the general population.^47^ As such to be considered for clinical use, new vectors must have a more favorable neutralization profile. This does not diminish the use of Ad5 in a vein graft gene therapy setting, but these findings may impact gene transfer settings where contact to the blood may be relevant, such as intravascular administration. The sera from 31% of patients tested in this study could neutralize Ad5, while none were able to reduce transduction with Ad49. Here we specifically used serum from a potential target population for vascular gene therapy, patients of a cardiovascular clinic. One previous study has also reported low levels of preexisting immunity to Ad49 in sera taken from four continents.^28^ Consistent with a previous study,^28^ there was no cross-reactive Ad5/Ad49-specific immunity observed in the serum cohort in this study.

Taken together, the data demonstrate that Ad49-based vectors have great potential for CABG gene therapy application in the clinic. Further investigation using Ad49 in preclinical CABG models is warranted.

## Supplementary Material

Supplemental data

## References

[1] Roger VL, Go AS, Lloyd-Jones DM, et al. Heart Disease and Stroke Statistics—2012 Update: a report from the American Heart Association. Circulation 2012;125:e2–e220 22179539 10.1161/CIR.0b013e31823ac046 PMC4440543

[2] Pacak CA, Byrne BJ AAV vectors for cardiac gene transfer: experimental tools and clinical opportunities. Mol Ther 2011;19:1582–1590 21792180 10.1038/mt.2011.124 PMC3182350

[3] Jessup M, Greenberg B, Mancini D, et al. Calcium Upregulation by Percutaneous Administration of Gene Therapy in Cardiac Disease (CUPID): a phase 2 trial of intracoronary gene therapy of sarcoplasmic reticulum Ca^2+^-ATPase in patients with advanced heart failure. Circulation 2011;124:304–313 21709064 10.1161/CIRCULATIONAHA.111.022889 PMC5843948

[4] Wan S, George SJ, Berry C, et al. Vein graft failure: current clinical practice and potential for gene therapeutics. Gene Ther 2012;19:630–636 22456326 10.1038/gt.2012.29

[5] Nicklin SA, Buening H, Dishart KL, et al. Efficient and selective AAV2-mediated gene transfer directed to human vascular endothelial cells. Mol Ther 2001;4:174–181 11545607 10.1006/mthe.2001.0424

[6] White SJ, Nicklin SA, Büning H, et al. Targeted gene delivery to vascular tissue in vivo by tropism-modified adeno-associated virus vectors. Circulation 2004;109:513–519 14732747 10.1161/01.CIR.0000109697.68832.5D

[7] George SJ, Wan S, Hu J, et al. Sustained reduction of vein graft neointima formation by *ex vivo* TIMP-3 gene therapy. Circulation 2011;124:S135–S142 21911803 10.1161/CIRCULATIONAHA.110.012732

[8] Akasaka Y, Ono I, Yamashita T, et al. Basic fibroblast growth factor promotes apoptosis and suppresses granulation tissue formation in acute incisional wounds. J Pathol 2004;203:710–720 15141387 10.1002/path.1574

[9] Varenne O, Pislaru S, Gillijns H, et al. Local adenovirus-mediated transfer of human endothelial nitric oxide synthase reduces luminal narrowing after coronary angioplasty in pigs. Circulation 1998;98:919–926 9738648 10.1161/01.cir.98.9.919

[10] Kibbe MR, Tzeng E, Gleixner SL, et al. Adenovirus-mediated gene transfer of human inducible nitric oxide synthase in porcine vein grafts inhibits intimal hyperplasia. J Vasc Surg 2001;34:156–165 11436090 10.1067/mva.2001.113983

[11] George SJ, Johnson JL, Angelini GD, et al. Adenovirus-mediated gene transfer of the human TIMP-1 gene inhibits smooth muscle cell migration and neointimal formation in human saphenous vein. Hum Gene Ther 1998;9:867–877 9581909 10.1089/hum.1998.9.6-867

[12] George SJ, Lloyd CT, Angelini GD, et al. Inhibition of late vein graft neointima formation in human and porcine models by adenovirus-mediated overexpression of tissue inhibitor of metalloproteinase-3. Circulation 2000;101:296–304 10645926 10.1161/01.cir.101.3.296

[13] George SJ, Angelini GD, Capogrossi MC, et al. Wild-type p53 gene transfer inhibits neointima formation in human saphenous vein by modulation of smooth muscle cell migration and induction of apoptosis. Gene Ther 2001;8:668–676 11406761 10.1038/sj.gt.3301431

[14] Kritz AB, Yu J, Wright PL, et al. In vivo modulation of Nogo-B attenuates neointima formation. Mol Ther 2008;16:1798–1804 18781142 10.1038/mt.2008.188 PMC4736735

[15] Lemarchand P, Jones M, Yamada I, et al. *In vivo* gene transfer and expression in normal uninjured blood vessels using replication-deficient recombinant adenovirus vectors. Circ Res 1993;72:1132–1138 8477524 10.1161/01.res.72.5.1132

[16] Rekhter MD, Simari RD, Work CW, et al. Gene transfer into normal and atherosclerotic human blood vessels. Circ Res 1998;82:1243–1252 9648720 10.1161/01.res.82.12.1243

[17] Parker A, White K, Lavery C, et al. Pseudotyping the adenovirus serotype 5 capsid with both the fibre and penton of serotype 35 enhances vascular smooth muscle cell transduction. Gene Ther 2013;20:1158–1164 24005577 10.1038/gt.2013.44 PMC3853367

[18] Vigl B, Zgraggen C, Rehman N, et al. Coxsackie- and adenovirus receptor (CAR) is expressed in lymphatic vessels in human skin and affects lymphatic endothelial cell function *in vitro*. Exp Cell Res 2009;315:336–347 19007771 10.1016/j.yexcr.2008.10.020

[19] Sumida SM, Truitt DM, Lemckert AA, et al. Neutralizing antibodies to adenovirus serotype 5 vaccine vectors are directed primarily against the adenovirus hexon protein. J Immunol 2005;174:7179–7185 15905562 10.4049/jimmunol.174.11.7179

[20] Bauer U, Flunker G, Bruss K, et al. Detection of antibodies against adenovirus protein IX, fiber, and hexon in human sera by immunoblot assay. J Clin Microbiol 2005;43:4426–4433 16145087 10.1128/JCM.43.9.4426-4433.2005 PMC1234141

[21] Kuriyama S, Tominaga K, Kikukawa M, et al. Inhibitory effects of human sera on adenovirus-mediated gene transfer into rat liver. Anticancer Res 1998;18:2345–2351 9703877

[22] White KM, Alba R, Parker AL, et al. Assessment of a novel, capsid-modified adenovirus with an improved vascular gene transfer profile. J Cardiothoracic Surg 2013;8:183 10.1186/1749-8090-8-183 PMC3751082 23937994

[23] Gaggar A, Shayakhmetov DM, Lieber A CD46 is a cellular receptor for group B adenoviruses. Nat Med 2003;9:1408–1412 14566335 10.1038/nm952

[24] Abbink P, Lemckert AaC, Ewald BA, et al. Comparative seroprevalence and immunogenicity of six rare serotype recombinant adenovirus vaccine vectors from subgroups B and D. J Virol 2007;81:4654–4663 17329340 10.1128/JVI.02696-06 PMC1900173

[25] Arnberg N, Kidd AH, Edlund K, et al. Initial interactions of subgenus D adenoviruses with A549 cellular receptors: sialic acid versus alpha(v) integrins. J Virol 2000;74:7691–7693 10906228 10.1128/jvi.74.16.7691-7693.2000 PMC112295

[26] Fallaux FJ, Bout A, Van Der Velde I, et al. New helper cells and matched early region 1-deleted adenovirus vectors prevent generation of replication-competent adenoviruses. Hum Gene Ther 1998;9:1909–1917 9741429 10.1089/hum.1998.9.13-1909

[27] Southgate K, Newby AC Serum-induced proliferation of rabbit aortic smooth muscle cells from the contractile state is inhibited by 8-Br-CAMP but not 8-Br-cGMP. Atherosclerosis 1990;82:113–123 2163252 10.1016/0021-9150(90)90150-h

[28] Lemckert AA, Grimbergen J, Smits S, et al. Generation of a novel replication-incompetent adenoviral vector derived from human adenovirus type 49: manufacture on PER.C6 cells, tropism and immunogenicity. J Gen Virol 2006;87:2891–2899 16963747 10.1099/vir.0.82079-0

[29] Vogels R, Zuijdgeest D, Van Rijnsoever R, et al. Replication-deficient human adenovirus type 35 vectors for gene transfer and vaccination: efficient human cell infection and bypass of preexisting adenovirus immunity. J Virol 2003;77:8263–8271 12857895 10.1128/JVI.77.15.8263-8271.2003 PMC165227

[30] Alba R, Baker A, Nicklin S Vector systems for prenatal gene therapy: principles of adenovirus design and production. In: Prenatal Gene Therapy. Coutelle C, Waddington SN, eds. (Humana Press, New York, NY). 2012; pp. 55–84 10.1007/978-1-61779-873-3_4 22648768

[31] Parker AL, Waddington SN, Buckley SMK, et al. Effect of neutralizing sera on factor X-mediated adenovirus serotype 5 gene transfer. J Virol 2009;83:479–483 18945780 10.1128/JVI.01878-08 PMC2612319

[32] Thorner AR, Vogels R, Kaspers J, et al. Age dependence of adenovirus-specific neutralizing antibody titers in individuals from Sub-Saharan Africa. J Clin Microbiol 2006;44:3781–3783 17021110 10.1128/JCM.01249-06 PMC1594810

[33] Jooss K, Chirmule N Immunity to adenovirus and adeno-associated viral vectors: implications for gene therapy. Gene Ther 2003;10:955–963 12756416 10.1038/sj.gt.3302037

[34] Sprangers MC, Lakhai W, Koudstaal W, et al. Quantifying adenovirus-neutralizing antibodies by luciferase transgene detection: addressing preexisting immunity to vaccine and gene therapy vectors. J Clin Microbiol 2003;41:5046–5052 14605137 10.1128/JCM.41.11.5046-5052.2003 PMC262545

[35] Wickham TJ, Mathias P, Cheresh DA, et al. Integrins αvβ3 and αvβ5 promote adenovirus internalization but not virus attachment. Cell 1993;73:309–319 8477447 10.1016/0092-8674(93)90231-e

[36] Roelvink PW, Mi Lee G, Einfeld DA, et al. Identification of a conserved receptor-binding site on the fiber proteins of CAR-recognizing adenoviridae. Science 1999;286:1568–1571 10567265 10.1126/science.286.5444.1568

[37] Campbell JH, Kocher O, Skalli O, et al. Cytodifferentiation and expression of alpha-smooth muscle actin mRNA and protein during primary culture of aortic smooth muscle cells. Correlation with cell density and proliferative state. Arterioscler Thromb Vasc Biol 1989;9:633–643 10.1161/01.atv.9.5.633 2675809

[38] Verma S, Lovren F, Pan Y, et al. Pedicled no-touch saphenous vein graft harvest limits vascular smooth muscle cell activation: the PATENT saphenous vein graft study. Eur J Cardio Thorac Surg 2014;45:717–725 10.1093/ejcts/ezt560 24327455

[39] Kipshidze N, Dangas G, Tsapenko M, et al. Role of the endothelium in modulating neointimal formation: vasculoprotective approaches to attenuate restenosis after percutaneous coronary interventions. J Am Coll Cardiol 2004;44:733–739 15312851 10.1016/j.jacc.2004.04.048

[40] Schulick AH, Dong G, Newman KD, et al. Endothelium-specific in vivo gene transfer. Circ Res 1995;77:475–485 7641320 10.1161/01.res.77.3.475

[41] Newman KD, Dunn PF, Owens JW, et al. Adenovirus-mediated gene transfer into normal rabbit arteries results in prolonged vascular cell activation, inflammation, and neointimal hyperplasia. J Clin Invest 1995;96:2955–2965 8675667 10.1172/JCI118367 PMC186007

[42] Von Der Leyen HE, Gibbons GH, Morishita R, et al. Gene therapy inhibiting neointimal vascular lesion: *in vivo* transfer of endothelial cell nitric oxide synthase gene. Proc Natl Acad Sci USA 1995;92:1137–1141 7532305 10.1073/pnas.92.4.1137 PMC42653

[43] Rutanen J, Turunen AM, Teittinen M, et al. Gene transfer using the mature form of VEGF-D reduces neointimal thickening through nitric oxide-dependent mechanism. Gene Ther 2005;12:980–987 15759018 10.1038/sj.gt.3302489

[44] Patel VI, Daniel S, Longo CR, et al. A20, a modulator of smooth muscle cell proliferation and apoptosis, prevents and induces regression of neointimal hyperplasia. FASEB J 2006;20:1418–1430 16816117 10.1096/fj.05-4981com

[45] Gupta R, Tongers J, Losordo DW Human studies of angiogenic gene therapy. Circ Res 2009;105:724–736 19815827 10.1161/CIRCRESAHA.109.200386 PMC2770893

[46] Jaski BE, Jessup ML, Mancini DM, et al. Calcium Upregulation by Percutaneous Administration of Gene Therapy in Cardiac Disease (CUPID Trial), a first-in-human phase 1/2 clinical trial. J Card Fail 2009;15:171–181 19327618 10.1016/j.cardfail.2009.01.013 PMC2752875

[47] Mast TC, Kierstead L, Gupta SB, et al. International epidemiology of human pre-existing adenovirus (Ad) type-5, type-6, type-26 and type-36 neutralizing antibodies: correlates of high Ad5 titers and implications for potential HIV vaccine trials. Vaccine 2010;28:950–957 19925902 10.1016/j.vaccine.2009.10.145

