# Supplementary material for: Efficient Transduction of Primary Vascular Cells by the Rare Adenovirus Serotype 49 Vector
Source: Hum Gene Ther. 2015 Mar 11;26(5):312–9. doi: 10.1089/hum.2015.019 (PMC4442572; doi:10.1089/hum.2015.019)
Supplement: Supplemental data [file Supp_Figure1.pdf]

Supplementary Data

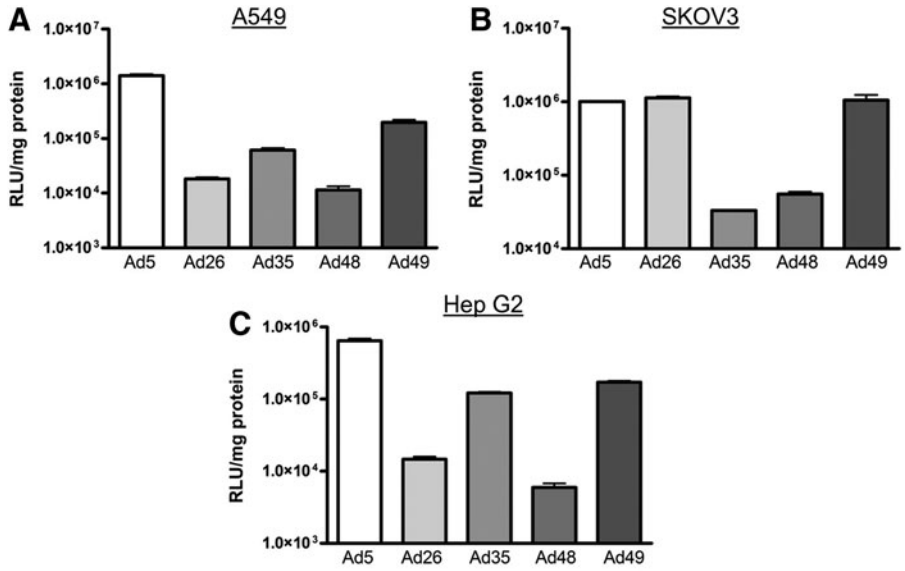

SUPPLEMENTARY FIG. S1. Transduction of cell lines. A549 (**A**), SKOV3 (**B**), and Hep G2 (**C**) cells were transduced with 10,000 vp/cell of Ad for 3 hr at 37°C. Cells were cultured for a further 48 hr before analysis of luciferase transgene expression was performed. Data are mean + SE.
